# Modular co-organization of functional connectivity and scale-free dynamics in the human brain

**DOI:** 10.1162/NETN_a_00008

**Published:** 2017-06-01

**Authors:** Alexander Zhigalov, Gabriele Arnulfo, Lino Nobili, Satu Palva, J. Matias Palva

**Affiliations:** Neuroscience Center, University of Helsinki, Finland; BioMag laboratory, HUS Medical Imaging Center, Helsinki University Central Hospital, Finland; Department of Computer Science, University of Helsinki, Finland; Department of Informatics, Bioengineering, Robotics and System Engineering, University of Genova, Italy; Claudio Munari Epilepsy Surgery Centre, Niguarda Hospital, Italy

**Keywords:** Scale-free dynamics, Functional connectome, Modular networks, Neuronal avalanches, Long-range temporal correlations

## Abstract

Scale-free neuronal dynamics and interareal correlations are emergent characteristics of spontaneous brain activity. How such dynamics and the anatomical patterns of neuronal connectivity are mutually related in brain networks has, however, remained unclear. We addressed this relationship by quantifying the network colocalization of scale-free neuronal activity—both neuronal avalanches and long-range temporal correlations (LRTCs)—and functional connectivity (FC) by means of intracranial and noninvasive human resting-state electrophysiological recordings. We found frequency-specific colocalization of scale-free dynamics and FC so that the interareal couplings of LRTCs and the propagation of neuronal avalanches were most pronounced in the predominant pathways of FC. Several control analyses and the frequency specificity of network colocalization showed that the results were not trivial by-products of either brain dynamics or our analysis approach. Crucially, scale-free neuronal dynamics and connectivity also had colocalized modular structures at multiple levels of network organization, suggesting that modules of FC would be endowed with partially independent dynamic states. These findings thus suggest that FC and scale-free dynamics—hence, putatively, neuronal criticality as well—coemerge in a hierarchically modular structure in which the modules are characterized by dense connectivity, avalanche propagation, and shared dynamic states.

## Author Summary

The framework of *criticality* has been suggested to explain the scale-free dynamics of neuronal activity in complex interaction networks. However, the in vivo relationship between scale-free dynamics and functional connectivity (FC) has remained unclear. We used human intracranial and noninvasive electrophysiological measurements to map scale-free dynamics and connectivity. We found that the propagation of fast activity avalanches and the interareal coupling of slow, long-range temporal correlations—two key forms of scale-free neuronal dynamics—were nontrivially colocalized with the strongest functional connections. Most importantly, scale-free dynamics and FC exhibited similar modular network structures. FC and scale-free dynamics, and possibly also neuronal criticality, appear to co-emerge in a modular architecture in which the modules are characterized internally by shared dynamic states, avalanche propagation, and dense functional connectivity.

The human connectome is a comprehensive map of how brain regions are mutually connected and is fundamentally important for understanding neuronal communication and brain dynamics in both health and disease (Fornito, Zalesky, & Breakspear, 2015). Although functional connectivity (FC) in the human brain was initially characterized as a stationary network (Raichle, 2009), recent empirical (Zalesky, Fornito, Cocchi, Gollo, & Breakspear, 2014) and theoretical (Deco, Jirsa, & McIntosh, 2013) studies have complemented this view by revealing considerable connectivity pattern fluctuations that are systematic beyond simply representing noise in weak stationary connectivity (Hansen, Battaglia, Spiegler, Deco, & Jirsa, 2015; Mitra, Snyder, Blazey, & Raichle, 2015). Such dynamic connectivity has been observed at a range of temporal scales from milliseconds to minutes (Larson-Prior et al., 2013). It has, nevertheless, remained unclear how these static and dynamic patterns of FC are related to the actual dynamical states of the brain and to the statistical patterns of activity propagation associated with this scale-free dynamics (Kopell, Gritton, Whittington, & Kramer, 2014).

The ubiquity of scale-free dynamics in neuronal activity suggests that neuronal systems could operate at a critical state (Chialvo, 2010). Critical neuronal dynamics are characterized, for instance, by the power-law scaling of neuronal activity avalanches at fast (10^−3^ to 10^−1^ s) timescales (Beggs & Plenz, 2003), as well as by power-law long-range temporal correlations (LRTCs) in neuronal fluctuations at slow (10^1^ to 10^3^ s) timescales (Linkenkaer-Hansen, Nikouline, Palva, & Ilmoniemi, 2001). However, the hierarchically modular rather than homogeneous structural connectivity of the brain may expand our concept of criticality from the system operating near a critical point into the system operating in an extended critical region or, more specifically, in a Griffiths phase (Hilgetag & Hutt, 2014; Moretti & Munoz, 2013). On the other hand, it is important to note that several kinds of noncritical processes are also associated with power-law LRTCs; hence, observations of scale-free dynamics do not, per se, indicate criticality (see, e.g., E. J. Friedman & Landsberg, 2013; Schwab, Nemenman, & Mehta, 2014).

Whether scale-free brain dynamics and/or neuronal criticality are related to FC has received attention only recently (Ciuciu, Abry, & He, 2014). Avalanche-like large-amplitude propagating patterns in BOLD signals have a connectivity structure similar to that of FC (Tagliazucchi, Balenzuela, Fraiman, & Chialvo, 2012). In the same vein, slow amplitude fluctuations of neuronal oscillations are characterized by both LRTCs (Linkenkaer-Hansen et al., 2001) and long-range interareal correlations with neuroanatomical structures similar to those of fMRI resting-state networks (Brookes et al., 2011). Hence, these converging, albeit indirect, relationships suggest that critical dynamics and the functional connectome could be linked, but in a manner that hitherto has been unclear. Recent computational-modeling studies have further elucidated this link by suggesting, on the one hand, that the modular network architecture of resting-state FC emerges specifically when the system is poised at a critical state (Haimovici, Tagliazucchi, Balenzuela, & Chialvo, 2013) and, on the other, that avalanches in a modular system propagate preferentially within connectivity modules (Russo, Herrmann, & de Arcangelis, 2014).

We advance here two hypotheses. First, we posit that neuronal avalanches propagate preferentially along the predominant pathways of FC and within the modules of the functional connectome. Second, we suggest that these modules are also endowed with partially independent dynamic states, which are reflected in stronger interareal correlations of LRTC exponents within the modules than between them. To test these hypotheses, we assessed the relationship between scale-free dynamics and FC by using intracranial stereotactic electroencephalography (SEEG) and magnetoencephalography (MEG). To ensure that the hypotheses were testable, we also performed several lines of control analyses to establish that the indices of dynamics and connectivity could be estimated independently. We characterized scale-free dynamics by connectomes of avalanche propagation patterns and interareal correlations in the LRTC scaling exponents, and compared the connection strengths and modular organizations in these connectomes with those in connectomes of FC measured by the phase synchronization (Vinck, Oostenveld, van Wingerden, Battaglia, & Pennartz, 2011) and amplitude correlations (Brookes, Woolrich, & Barnes, 2012) of ongoing neuronal oscillations. We show that both neural dynamics and FC have, in a frequency-specific and nontrivial manner, similar constellations of strongest connections and shared modular structures, which provides empirical evidence that neuronal connectivity and scale-free dynamics are intimately linked.

## RESULTS

**Figure 1.**
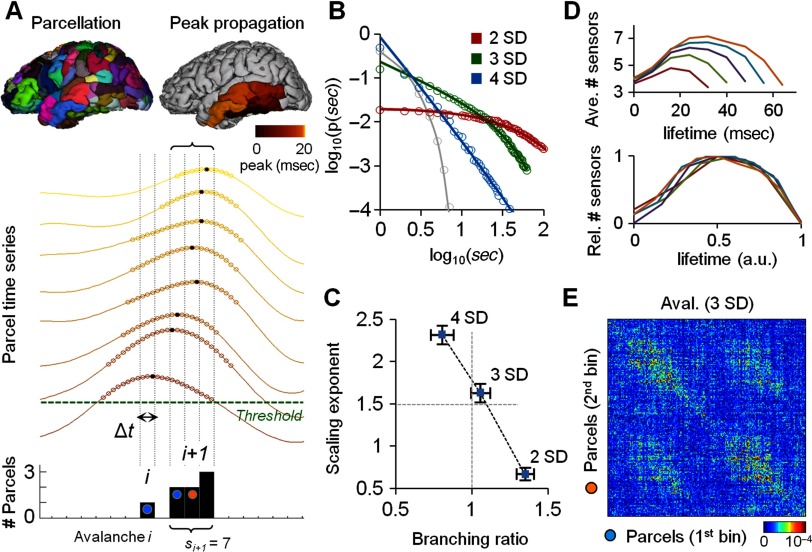
Power-law scaling neuronal activity avalanches reveal signatures of critical dynamics. (A) We used a parcellation (top panel) comprising 219 neuroanatomically labeled cortical parcels in an individual cortical anatomy as the basis for all analyses. Neuronal avalanches were detected in multichannel MEG (colored lines in middle panel; for simplicity, only the MEG data are plotted in this figure) and SEEG recordings, where avalanches (black bars at bottom) were defined as sets of uninterrupted time bins containing one or more suprathreshold peaks (black dots). The avalanche time series—that is, the numbers of events per time bin—are depicted with the black bars. The first and second time bins used in the avalanche propagation connectome are marked with blue and orange dots, respectively. (B) Example of avalanche size distributions for the different thresholds and a time bin width of 8 ms in the MEG data. The distributions are approximated well by truncated power laws for the thresholds of two (red line), three (green line), and four (blue line) *SD*s. In contrast, the avalanche sizes for surrogate data follow an exponential distribution (gray line). (C) Mean power-law scaling exponents and branching ratios for the three thresholds (error bars indicate the standard deviations for each mean) in the MEG data. (D) Avalanches exhibit a universal scaling function, a hallmark of a critical process; graphs show the average shapes of the neuronal avalanches associated with different lifetimes (upper panel) and the collapse after normalization of the lifetimes (lower panel). (E) MEG group-level neuronal avalanche propagation connectomes represent the probabilities of avalanche propagation (color scale) from each parcel to each other parcel for the threshold of three *SD*s.

In our MEG and SEEG data, a *neuronal avalanche* was defined as a set of suprathreshold peaks in waves of broadband (1–120 Hz) ongoing activity occurring in consecutive time bins (Figure 1A). The statistical properties of avalanches can be described by the distributions of their sizes—that is, the total numbers of peaks in each avalanche—and lifetimes—that is, the durations of the avalanches. In this study we used time bin widths of 8 and 16 ms for the MEG and SEEG data, respectively, and thresholds of two, three, and four standard deviations (*SD*s) to characterize the propagation of neuronal avalanches in different scaling regimes (Zhigalov, Arnulfo, Nobili, Palva, & Palva, 2015). To evaluate the overall statistical nature of these avalanches, we first assessed the goodness of fit between the size distribution data and a truncated power-law model by computing the Kolmogorov–Smirnov distance (*D*_KS_). The largest *D*_KS_ for the different thresholds was 0.04 (*p* > 0.85) suggesting that the distributions were approximated well by the truncated power-law function (Figure 1B). The scaling exponents of the avalanche sizes were estimated using the maximum-likelihood approach (see Materials and Methods). We found similar mean power-law scaling exponents in the MEG and SEEG data, with values close to 1.5 at the three-*SD* threshold (Figure 1C). We also assessed the avalanche branching ratios at different thresholds and observed values close to 1 at three *SD*s in both the MEG and SEEG data. The scaling exponent of 1.5 and branching ratio of 1 (Figure 1C) correspond to a critical branching process and are well in line with a body of prior observations on neuronal avalanche dynamics (D. Plenz, 2012).

To corroborate the truncated power-law fitting of the distributions of avalanche sizes, we applied a likelihood ratio test to compare the truncated power-law model with an exponential model. The results showed that the truncated power-law model outperformed the exponential model (*p* < 0.001, log-likelihood ratio test) for all subjects and thresholds.

The distributions of the avalanche lifetimes were biased by the numbers of samples and were less robust than the size distributions in individual subjects. To assess the lifetime distributions, we thus concatenated avalanches across subjects. Consistent with the prior experimental and theoretical literature, the power-law exponents of the avalanche lifetimes were near 2 at the three-*SD* threshold, where a size distribution exponent of 1.5 was found (Zhigalov, Arnulfo, Nobili, Palva, & Palva, 2017, Figure S1).

Finally, we assessed the scaling function for the shapes of the neuronal avalanches (Figure 1D), by averaging avalanches within ranges of lifetimes (Figure 1D, upper panel) and then rescaling the functions by normalizing the lifetimes (Figure 1D, lower panel). At rescaling, a collapse of the avalanche shapes was observed, which is again well in line with previous studies (Beggs & Timme, 2012; N. Friedman et al., 2012) and further supports the hypothesis that the neuronal avalanches observed here arose from a critical process.

We characterized the spatial-propagation patterns of neuronal avalanches by compiling a connectome of the empirical probabilities with which the activity peaks of the cortical parcels in the first time bin were followed by activity peaks of the parcels in the second time bin (Figure 1E and Zhigalov et al., 2017, Figure S2; see Materials and Methods).

### Construction of Functional Connectivity and LRTC Connectomes

**Figure 2.**
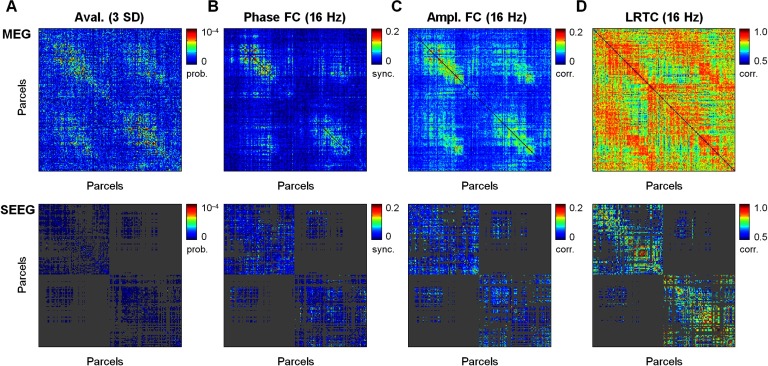
Avalanche propagation and the connectomes of interareal relationships in local LRTCs have overall structures similar to those of the functional connectomes of phase synchronization and amplitude correlations in both MEG and SEEG. (A) Avalanche propagation connectomes represent the probabilities (color scale) of avalanche propagation from parcel to parcel. (B) Phase-synchronization-based functional connectome at 16 Hz (color scale, showing mean weighted phase-lag indices). (C) Amplitude-correlation-based functional connectome at 16 Hz (color scale, showing mean Pearson correlation coefficients of the orthogonalized amplitude time series). (D) Connectome of interareal correlations in the local LRTCs at 16 Hz (color scale, showing Pearson correlation coefficients across subjects). The top and bottom panels represent the MEG and SEEG connectomes, respectively. Gray areas in the adjacency matrices (bottom panels) indicate parcel–parcel connections not sampled in the present SEEG subject cohort.

We hypothesized that the neuronal avalanches observed in MEG and SEEG (Figure 2A) would propagate preferentially between brain areas coupled by FC. To test this hypothesis, we measured electrophysiological FC by means of the pairwise phase synchronization and amplitude correlations (Figures 2B and 2C) of narrow-band neuronal oscillations. Furthermore, to assess the interareal relationships of scale-free dynamics per se—that is, to measure how local dynamical states were correlated among all cortical regions—we obtained LRTC connectomes (Figure 2D) by correlating across subjects the LRTC scaling exponents of orthogonalized amplitude time series for all pairs of parcels. The LRTC exponents are proportional to the system’s proximity to the critical point (Poil, Hardstone, Mansvelder, & Linkenkaer-Hansen, 2012), and hence, if brains behaved like a “single” near-critical system, this connectome should be uniform. However, already a visual inspection of the LRTC connectome (see Figure 2D) showed that, like the other connectomes, it was highly nonhomogeneous, and although many brain areas were significantly correlated in both MEG (*r* > 0.54, *p* < 0.05, Pearson’s correlation test) and SEEG (*r* > 0.39, *p* < 0.05, Pearson’s correlation test), also a considerable fraction of regions were below these nominal significance thresholds. Hence, at least among some subsets of cortical areas, the local dynamics were only weakly correlated.

### Avalanche Propagation Has Connectivity Patterns Similar to Those of FC

**Figure 3.**
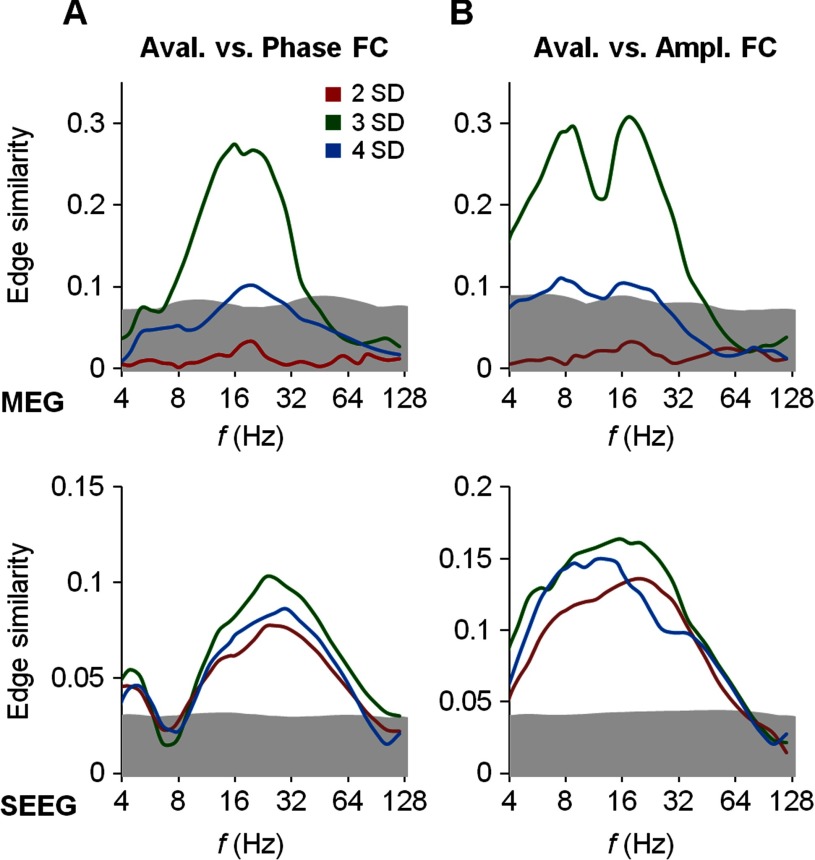
Avalanche propagation and functional connectivity (both phase and amplitude FC) are colocalized in both MEG and SEEG. (A, B) The colocalization of broadband avalanche propagation at two (red line), three (green line), and four (blue line) *SD*s and of the narrow-band phase-synchronization (A) or amplitude-correlation (B) connectomes was measured with edge similarity (see Materials and Methods) for each frequency band in the MEG (top panels) and SEEG (bottom panels) data. The gray-shaded areas indicate the surrogate-data-derived (see Materials and Methods) confidence interval of 99.9%, corresponding to a significance criterion of *p* < 0.001.

To test the hypothesis of colocalized avalanche propagation pathways and FC, we first measured the connection-level similarity—that is, the *edge similarity*—of avalanche propagation with phase synchronization and amplitude correlations by means of the Pearson correlations of these connectomes (see Materials and Methods). For phase-correlation-based FC, the results showed that the edge similarity was highly significant (well above the 99.9% confidence intervals of the surrogate data, corresponding to uncorrected *p* < 0.001) between avalanche propagation at three *SD*s and phase synchronization. This relationship was found in a limited range of frequencies around the *α* and *β* bands (i.e., 8–14 Hz and 15–30 Hz, respectively; Figure 3A, green lines), with peaks at around 16 Hz in MEG and at around 32 Hz in SEEG. The similarity between phase correlations and avalanche propagation at both two and four *SD*s was much weaker (*p* < 0.001, Wilcoxon test) in both the *α* and *β* bands than at three *SD*s, and significant only at four *SD*s in the *β* band (Figure 3A, blue lines) in MEG. The edge similarity between avalanche propagation and the phase connectomes was also highly significant in SEEG (Figure 3A), but the differences between thresholds were not significant in SEEG (*p* > 0.26, Wilcoxon test).

Similar to the phase-correlation FC, amplitude-correlation-based FC was also significantly correlated with avalanche propagation at three *SD*s for frequencies up to ∼30 Hz in MEG and up to ∼60 Hz in SEEG (Figure 3B, green lines). The spectral profiles of these similarities were, however, distinct from those for phase FC in extending to the lowest frequencies (4−8 Hz), even clearly peaking at 8 Hz (in MEG). This suggests that the amplitudes but not the phase couplings of 4- to 8-Hz oscillations are associated with the propagation pathways of broadband avalanches. The similarity was again significantly greater for avalanche propagation at three *SD*s than at two or four *SD*s (Figure 3B) across the entire range of frequencies in MEG (*p* < 0.001, Wilcoxon test), whereas these differences in SEEG were not significant. These data thus suggest that neuronal avalanches indeed propagate preferentially between brain areas that are coupled by phase synchronization and/or amplitude correlations.

### Avalanche Propagation Is Phenomenologically Distinct and Analysis-Wise Separable From Phase Synchronization and Amplitude Correlations

**Figure 4.**
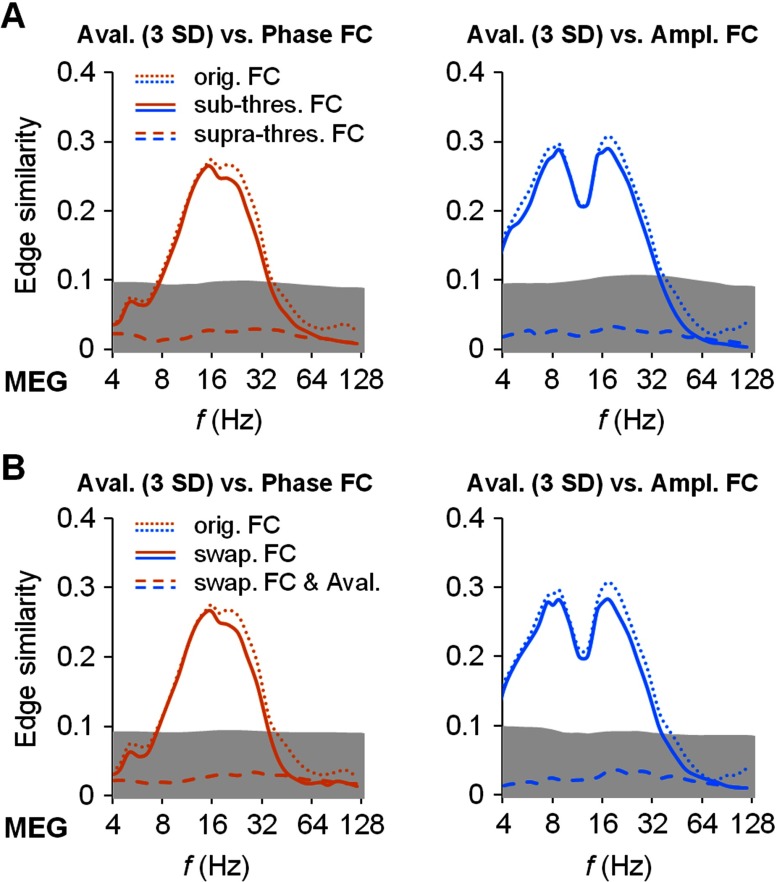
Avalanche propagation is phenomenologically distinct and analysis-wise separable from both phase synchronization and amplitude correlations. (A) Edge similarity of the avalanche connectome at three *SD*s with the phase-synchronization (left panel) and amplitude-correlation (right panel) connectomes estimated for subthreshold (solid lines) and suprathreshold (dashed line) segments. The result that the subthreshold similarity (solid lines) is close to the original similarity between avalanche propagation at three *SD*s and FC (dotted lines; see Figures 3A and3B) shows that the avalanche segments in the data are irrelevant to the estimation of FC. The suprathreshold similarity (dashed lines) being well inside the 99.9% confidence limits (gray areas) indicates that FC during the avalanche segments does not have a structure similar to that of avalanche propagation. (B) Edge similarity of the avalanche connectome at three *SD*s with the phase-synchronization (left panel) and amplitude-correlation (right panel) connectomes (solid lines), estimated from data in which all suprathreshold segments were swapped across random pairs of parcels prior to estimation of the phase/amplitude connectomes. The result that this similarity is again close to the original similarity estimates (dotted lines) indicates that the avalanches have a negligible contribution to the FC estimates. When the reorganized avalanches were detected from the same data (dashed lines), their similarity was well below the confidence limits (gray area).

To corroborate that the avalanche propagation and phase or amplitude connectomes can be estimated independently, and hence to ensure that the hypotheses tested above were falsifiable, we performed two types of control analyses (see Materials and Methods). In the first approach, we deleted from the narrow-band data all data segments in which the broadband amplitude exceeded the avalanche detection threshold—that is, all data used in the avalanche analyses—and recomputed the phase and amplitude connectomes. Evaluation of their similarity with the original avalanche propagation connectome revealed high similarity that was not significantly different from what we had obtained with the uncut data (*p* > 0.99, KS test; Figure 4A, solid lines). Reversing this analysis, we estimated the phase-synchronization and amplitude-correlation connectomes by only using the suprathreshold data. Here, the similarity of the suprathreshold functional connectomes with the original avalanche propagation connectome (Figure 4A, dashed lines) was at chance level. These analyses thus show that the similarity of FC and avalanche propagation does not arise from temporally colocalized time windows in the data: avalanches neither bias the quantification of FC nor are trivially produced by concurrent FC.

To corroborate these insights, in the second approach, we swapped all the time windows of suprathreshold avalanche segments between random parcel pairs, and thereby systematically reordered the avalanche propagation connectome. We then refiltered the data and recomputing the phase-synchronization and amplitude-correlation connectomes. Their similarity with the original avalanche propagation connectome did not decrease significantly much below the original values (*p* > 0.99, KS test; Figure 4B, solid lines). On the other hand, the reordered avalanche propagation connectome was uncorrelated with the functional connectomes estimated from the same reordered data (Figure 4B, dashed lines). Although this result was not surprising, in the sense that the original correlation of FC and avalanches was broken by reorganizing the avalanches, this analysis confirmed the first one and showed conclusively that the bias that the avalanche segments impose on FC estimates from the same data is negligible, and hence that the avalanches and FC can be estimated independently.

These data, together with the prior findings of (i) avalanche–FC correlations being distinct for amplitude and for phase FC; (ii) avalanche–FC correlations being spectrally limited, whereas FC is not (see below and in Figure 6); and (iii) avalanches at two *SD*s not being more similar to FC than at three *SD*s, thus strongly suggest that the broadband avalanches are neuro physiologically and phenomenologically distinct from the multitude of phase-/amplitude-coupled narrow-band oscillations.

### Interareal Correlations in LRTCs Are Similar to Those of FC

**Figure 5.**
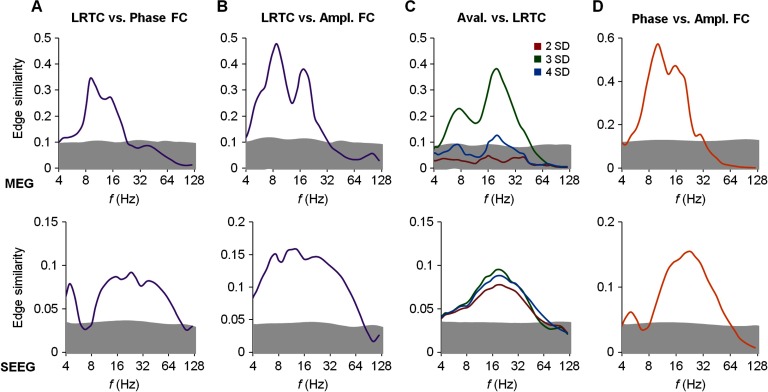
LRTC and functional connectivity (both phase and amplitude FC) are colocalized in both the MEG and SEEG data. (A, B) The colocalization of the LRTC and narrow-band phase-synchronization (A) and amplitude-correlation (B) connectomes was measured with edge similarity (see Materials and Methods) for each frequency band in the MEG (top panel) and SEEG (bottom panel) data. (C) Edge similarity between avalanche propagation at two (red lines), three (green lines), and four (blue lines) *SD*s and the LRTC connectomes. (D) Edge similarity between the phase and amplitude connectomes. The gray-shaded areas indicate the surrogate-data-derived (see Materials and Methods) confidence interval of 99.9%, corresponding to a significance criterion of *p* < 0.001.

We then assessed how the interareal relationships in local LRTCs were correlated with the patterns of FC. To this end, we first estimated the edge similarity between the LRTC and phase/amplitude connectomes and found robustly significant, but again band-limited, similarities (Figures 5A and 5B) with spectral profiles akin to those found for avalanche propagation (see Figures 3A and3B). Finally, the comparison of avalanche propagation at three *SD*s with the LRTC connectomes also revealed significant similarity in both the MEG and the SEEG data (Figure 5C). The similarity was significantly larger for the avalanche propagation at three than at two and four *SD*s in the MEG data (*p* < 0.001, Wilcoxon test). Moreover, the phase-synchronization and amplitude-correlation connectomes were clearly correlated in both MEG and SEEG (Figure 5D), with 16 and 32 Hz as the modal frequencies, respectively. Throughout these similarity estimates, and also in the mutual correlation of phase and amplitude FC, the frequency band of significant correlations extended to ∼60 Hz in SEEG and only to ∼30 Hz in MEG, which is likely attributable to the approximately millimeter versus approximately centimeter spatial scales across which SEEG and MEG measure coherent neuronal population activity.

These findings together thus show that scale-free brain dynamics, including both power-law-scaled, millisecond-range activity avalanches and LRTCs spanning hundreds of seconds, are intimately linked with the architecture of FC in human cortex.

### LRTCs of Phase Synchrony Show Strong Similarity to FC

The findings so far converged on the idea that two facets of critical dynamics, avalanche propagation and interareal coupling of local LRTCs, were colocalized with phase- and amplitude-correlation-based functional connectivity. To corroborate this finding with an independent index of critical dynamics, we estimated LRTCs in the momentary dynamics of phase synchrony with phase detrended fluctuation analysis (DFA; Botcharova, Farmer, & Berthouze, 2014) between all pairs of cortical parcels in the MEG data. The adjacency matrices of the phase DFA LRTC scaling exponents were thus directly comparable with those of FC. We found phase DFA and FC to be highly significantly similar for both phase and amplitude FC (well above the 99.9% confidence intervals) in the *α* and *β* frequency bands and to have profiles similar to those of the avalanche propagation and functional connectomes (Figure 3). These observations show that temporal correlations and spatiotemporal interactions are linked at different timescales, which consolidates the prior findings (see Figures 4 and 5) and overall suggests that the underlying system is poised at criticality.

### Alpha–Beta-Range Oscillations Form a Cortical Core for the Dynamics–Connectivity Association

The association of scale-free dynamics and FC was found in a relatively narrow frequency range around the *α* and *β* frequency bands. Does this reflect a “special” and nontrivial emergent phenomenon or a trivial “by-product” of the scale-free dynamics being automatically associated with connectivity?

**Figure 6.**
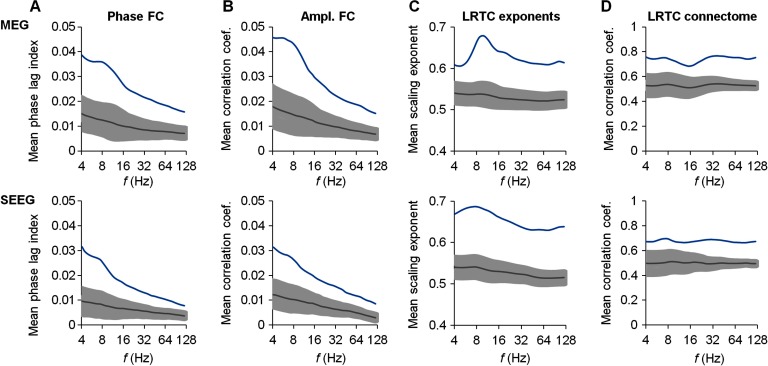
Highly significant FC and LRTCs are observed throughout the analyzed frequency range. (A, B) Mean connection strengths (blue lines) of the phase (A) and amplitude (B) connectomes as a function of frequency in the MEG (top panels) and SEEG (bottom panels) data. Gray lines indicate the corresponding mean values for the surrogate data, and the gray-shaded areas show the confidence limits of 99.9%. (C) LRTC scaling exponents (blue lines) averaged across all parcels peak at 10 Hz in the MEG (top panel) and at 8 Hz in the SEEG (bottom panel) data, respectively. (D) Mean connection strengths (blue lines) of the LRTC connectome as a function of frequency in the MEG (top panel) and the SEEG (bottom panel) data.

To address this question, we asked whether either FC or LRTCs were correspondingly more prevalent in this *α*/*β* range than in the surrounding bands. We first assessed the connection strengths of the phase-, amplitude-, and LRTC-correlation connectomes across the frequency range. For the phase and amplitude connectomes, the mean correlation strengths exhibited subtle peaks, at 9 Hz in MEG and at 7 Hz in SEEG, but overall they decreased monotonically as a function of frequency (Figures 6A and 6B) and remained well above the 99.9% confidence limits of the corresponding surrogate values throughout the studied frequency range. Both phase- and amplitude-based forms of FC thus appear robust for frequencies between 4 and 128 Hz. Similar averaging of the local LRTC scaling exponents (Figure 6C) showed that the LRTCs also peaked at around 10 Hz in MEG and at 8 Hz in SEEG, but again were well above the surrogate confidence limits from 4 to 128 Hz. Significant scale-free dynamics are thus not unique to any single frequency band. In the same vein, the interareal correlations of LRTC exponents were essentially constant and highly significant across the studied frequency range (Figure 6D). Hence, just as FC was a salient characteristic of all frequency bands, also scale-free dynamics and the interareal structure of LRTCs characterized the 8- to 32-Hz range over which most of the salient dynamics–FC coupling was found, as well as the 4- to 8-Hz and 32- to 128-Hz ranges over which the dynamics–FC correlations were much weaker or insignificant. Together with the previous analyses (see Figure 5), these results thus show that significant FC and scale-free dynamics in the human brain can be mutually coupled or uncoupled in a frequency-dependent manner, which indicates that this coupling is nontrivial and neither automatic nor epiphenomenal.

### Shared Modular Structures of Avalanche Propagation, LRTCs, and Functional Connectivity

The similarity between avalanche propagation pathways, interareal correlations of LRTCs, and functional connectomes indicated a significant connection-level colocalization of these networks. We addressed next whether scale-free dynamics and FC had similar modular structures in the MEG data. The SEEG data were excluded from this analysis because their sparse spatial coverage did not allow for robust detection of the functional modules.

**Figure 7.**
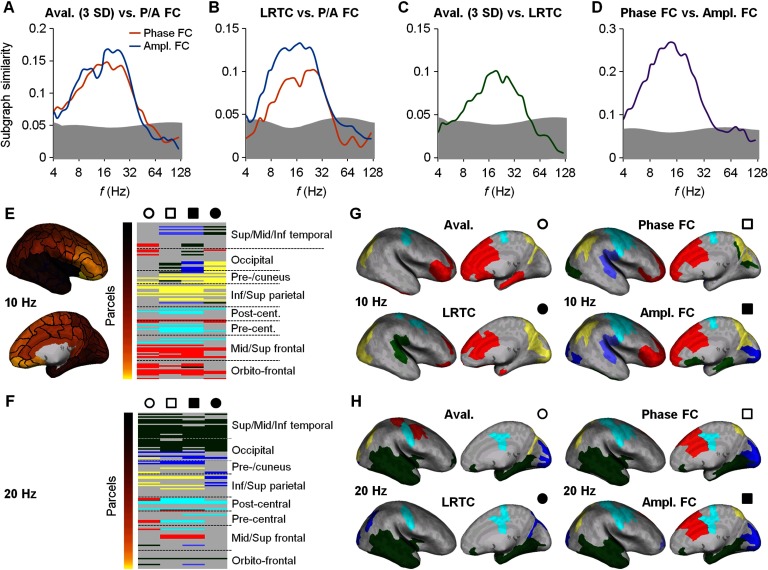
Neuronal criticality and functional connectivity (FC) have similar modular structures in the MEG data. (A) The colocalization of the modular structures in the connectomes was assessed with a subgraph similarity measure (see Materials and Methods). The modular structure of the avalanche propagation connectome at three *SD*s was highly significantly similar to the modular structures of the phase and amplitude connectomes from about 4 to 40 Hz. (B) The spectral profiles of subgraph similarity between LRTC and the phase/amplitude connectomes are comparable to those of the avalanche propagation connectome. (C) Subgraph similarity between the avalanche propagation connectome at three *SD*s and the LRTC connectome. (D) Subgraph similarity between the phase and amplitude connectomes. The shaded areas in all graphs indicate the 99.9% confidence intervals. (E, F) A large fraction of parcels have similar subgraph assignments among the avalanche propagation, phase, amplitude, and LRTC connectomes; hence, these connectomes exhibit similar modular structures at both 10 and 20 Hz. Lavender, green, blue, yellow, cyan, and red colors indicate the subgraph identities. The inflated brains illustrate the color codes for the parcels scale. (G, H) Neuroanatomical visualization of matching subgraphs (functional modules) in the avalanche propagation connectome and the phase, amplitude, and LRTC connectomes at 10 and 20 Hz, respectively. The circle and square symbols indicate correspondences between the subgraphs in the plane (E, F) and neuroanatomical (G, H) representations.

We identified network modules, *subgraphs*, by using agglomerative hierarchical clustering of the connectomes of scale-free dynamics and FC (see Figure 2), and then measured the similarity of these modular structures by quantifying the overlap among the subgraph assignments (see Materials and Methods). The results showed that the modular structures of both avalanche propagation at three *SD*s (Figure 7A) and the interareal correlations of LRTCs (Figure 7B) had highly significant subgraph similarities with both phase- and amplitude-based FC. This colocalization of network modules was found in a limited frequency range up to 40 Hz, with the greatest values again being between 8 and 30 Hz (Figures 7A and 7B). For completeness, we also measured the subgraph similarity between avalanche propagation at three *SD*s and LRTCs (Figure 7C), as well as between the levels of phase and amplitude FC (Figure 7D), and found that both were robust in the 8- to 32-Hz range. In this frequency range, scale-free dynamics and FC thus had both internally and mutually shared modular structures.

To identify the cortical regions that composed the shared functional modules in these connectomes, we used consensus mapping of the parcel-subgraph neighborhoods obtained with the hierarchical clustering (see Materials and Methods). This analysis showed that the most consistently shared modules were localized to sensorimotor, visual, temporal, and prefrontal areas (*p*s < 0.05, permutation test; see the cyan, blue, green, and red modules, respectively, in Figures 7E–7H).

## DISCUSSION

Criticality governs the spatiotemporal dynamics of numerous natural and artificial systems composed of large numbers of nonlinearly interacting elements. At the critical operating point, the system exhibits long-range correlations with power-law decay concurrently across all temporal and spatial scales (Bak, Tang, & Wiesenfeld, 1988; Chialvo, 2010). Brains have been proposed to operate near the critical point (Beggs & Plenz, 2003; Linkenkaer-Hansen et al., 2001) or in an extended critical region (Hilgetag & Hutt, 2014; Moretti & Munoz, 2013), which has fundamental implications for neurophysiological phenomena and neuronal information processing across the scales from neurons to brain systems. Neuronal criticality has primarily been operationalized with two hallmark phenomena, millisecond-range neuronal avalanches (D. Plenz, 2012) and long-range temporal correlations spanning hundreds of seconds (Linkenkaer-Hansen et al., 2001), although power-law characteristics have also been reported for several other forms of spatiotemporal brain dynamics (Kitzbichler, Smith, Christensen, & Bullmore, 2009). Conversely, although scale-free dynamics are an indisputable characteristic of brain activity, whether they arise from criticality or other mechanisms has remained a matter of debate (Touboul & Destexhe, 2010).

Much of our understanding of spatiotemporal brain dynamics has arisen from studies of hemodynamic and electrophysiological FC, which have shown that hierarchically modular architectures of neuronal interactions govern both resting-state brain activity (Mitra et al., 2015) and ongoing activity during task performance (de Pasquale et al., 2012). It is theoretically conceivable and predicted by computational models (Haimovici et al., 2013) that neuronal criticality and FC—concurrent, yet distinct and dissociable, network phenomena—would be related. However, very few prior experimental studies have aimed at elucidating this relationship (Pajevic & Plenz, 2009; Rubinov, Sporns, Thivierge, & Breakspear, 2011).

In this study, we sought to bridge this gap by quantifying the interareal relationships of scale-free dynamics and FC. Toward this end, we used both meso- and macro-scale electrophysiological recordings of human resting-state brain activity, with invasive SEEG (Arnulfo, Hirvonen, Nobili, Palva, & Palva, 2015) and noninvasive MEG (S. Palva & Palva, 2012), respectively. We first found that at the level of the strongest connections, avalanche propagation was well colocalized with both phase-synchronization-based and amplitude-correlation-based FC. To investigate how these interactions were coupled with scale-free dynamics, we estimated the interareal correlations of local LRTCs and found that these correlations were also colocalized with both avalanche propagation and FC. Scale-free neuronal dynamics and FC thus have a shared backbone of interareal relationships.

Both functional (Gallos, Makse, & Sigman, 2012; Meunier, Lambiotte, & Bullmore, 2010) and structural (Hagmann et al., 2008) connectivity have hierarchically modular network organizations. As the second step in this study, we assessed the modularity in our estimates of critical dynamics and found that both the propagation of neuronal avalanches and the interareal correlations of LRTCs exhibited modular structures, and that these structures were neuroanatomically similar to those of FC. This strongly suggests that neuronal communities characterized by strong internal phase synchronization and amplitude correlations are also characterized by preferentially internal avalanche propagation and correlated local LRTCs. Beyond linking scale-free dynamics and connectivity, this finding also indicates that the brain can be envisioned as a constellation of mutually coupled and hierarchically organized modules distributed to distinct neuroanatomical substrates and to different frequency bands, rather than as a homogeneous critical-state system.

Overall, these observations strongly suggest that FC and scale-free neuronal dynamics, and hence, putatively, neuronal criticality as well, are intimately related, which opens new avenues for studies aiming to understand the physiological interactions between these phenomena.

### Spatial Domains of Scale-Free Dynamics Revealed by Interareal LRTC Relationships

Neuronal criticality is often considered in the context of models or physical systems that have a relatively homogeneous spatial structure of their interacting elements (N. Friedman et al., 2012; D. Plenz & Thiagarajan, 2007). In contrast, human brains have a markedly nonhomogeneous and hierarchically modular large-scale structure (Gallos et al., 2012; Meunier et al., 2010), which theoretically has a major influence on the emergence of criticality in neuronal activity, by transforming the system from having a critical point into exhibiting a critical regime—that is, a Griffith’s phase (Hilgetag & Hutt, 2014; Moretti & Munoz, 2013). One key implication of modular structural connectivity is that it could allow for topologically distant modules of the network to have semi-independent dynamics. In this study, the finding that the interareal relationships of LRTC scaling exponents were clearly nonhomogeneous provides evidence for this theory and opens the possibility that different brain systems could have distinct operating points (or regimes).

We found also that both the interareal relationships and modular structures of neuronal avalanches and LRTCs had colocalized neuroanatomical bases. This finding complements prior studies that have shown that scale-free dynamics observed at the meso- and macro-scales with SEEG and MEG, respectively, are phenomenologically very similar (Zhigalov et al., 2015) and that the avalanche and LRTC scaling exponents are corrected at both spatial scales (J. M. Palva et al., 2013).

### Frequency Specificity in the Dynamics–Connectivity Association

The connectivity patterns of oscillations in and around the *α* (8–14 Hz) and *β* (15–30 Hz) bands have been suggested to provide a “cortical core” (de Pasquale et al., 2012) or “hubs” (Hipp, Hawellek, Corbetta, Siegel, & Engel, 2012) for neuronal integration and resting-state network dynamics. We found here that the strongest similarities between scale-free dynamics and FC were observed specifically in this frequency range. Likewise, the similarity between neuronal avalanches and LRTC was also maximal in this frequency range, and an even stronger similarity was found for both interareal phase synchronization and amplitude correlations (see Figure 5D). The *α* and *β* oscillations in the resting state thus appear to be central for both neuronal integration and the connectivity–dynamics association. Importantly, the lack of such association at the lower frequencies (4–7 Hz) was not explained by a lack of FC or by evidence for power-law scale-free dynamics therein, because highly significant FC as well as LRTCs were observed in this frequency range (see Figure 6). This shows that the association of FC and scale-free dynamics is neither trivial nor automatic in brain dynamics, but rather is a unique dynamic property of a subset of frequency bands in ongoing brain activity.

### Possible Confounders

Two sources of circularity could theoretically confound our analyses of the similarities between broadband avalanche propagation and narrow-band phase synchronization or amplitude correlations. As a conceptual confound, one may argue that the broadband avalanche events and the narrow-band oscillations actually reflect the dynamics of a single underlying neuronal process, which would lead to artificial or trivial colocalization of their connectomes. As a technical confound, the estimates of narrow-band FC could be biased by the putatively large-amplitude avalanche events, and conversely, the avalanche detection might pick up momentarily large-amplitude oscillations because both phenomena were estimated from the same data. Such “technical” cross-bias in the estimators of avalanche propagation and FC could render the estimators mutually and artificially correlated by design. However, control analyses (see Figure 4) showed that (i) the rare avalanche-like events in ongoing brain activity have a negligible impact on estimates of FC, and (ii) both avalanche propagation and FC can be estimated independently of each other. Thus, no technical circularity prevented our testing the hypothesized relationship between avalanches and connectivity. Moreover, because (iii) narrow-band FC during the avalanches was *uncorrelated* with the avalanche propagation estimated from the same data, the propagating broadband avalanche events appear to be distinct neurophysiological phenomena from the interareal phase or amplitude couplings of narrow-band oscillations. Finally, the finding that the avalanche propagation patterns detected at two *SD*s were less similar to FC than were those detected at three *SD*s constitutes strong evidence that the avalanche–connectivity relationship is not a trivial by-product of avalanches simply reflecting phase-lagged connectivity, because if this were the case, the two-*SD* avalanches should be closer in similarity to the FC estimates (which effectively correspond to the “0-*SD*” data) than the avalanches at three *SD*s.

### Clinical Implications

Several lines of evidence have shown that abnormalities in neuronal scaling laws are biomarkers of a number of brain diseases. In major depressive disorder, the LRTC scaling exponents for central/frontal theta oscillations are lower than those among healthy controls, whereas posterior *α* oscillations exhibit no abnormalities (Linkenkaer-Hansen et al., 2005). In schizophrenia, the LRTC scaling exponents are suppressed in both the *α* and *β* bands over parietotemporal areas (Nikulin, Jonsson, & Brismar, 2012). Similarly, in autism spectrum disorders (Lai et al., 2010) and Alzheimer’s disease (Montez et al., 2009), lower-than-normal LRTC scaling exponents are a robust characteristic. Conversely, in epileptic patients, the epileptogenic zone exhibits greater *β*-band LRTC exponents than do the surrounding cortical regions (Monto, Vanhatalo, Holmes, & Palva, 2007). These findings are well in line with our discovery that different brain systems are capable of operating concurrently in distinct dynamic regimes. The intriguing, and possibly functionally essential, implication is that abnormal dynamics affecting only specific brain systems and/or frequency bands could be predictive of corresponding pathological states and mental symptoms. Neuroanatomical specificity in critical dynamics is also salient in the relationship between healthy neuronal and behavioral fluctuations, in which neuronal criticality only in well-delineated brain systems is predictive of behavioral scaling laws (J. M. Palva et al., 2013). Future studies should thus examine the possibility of modulating cortically well-localized dynamics noninvasively—for example, by using neurofeedback (Ros et al., 2016; Zhigalov, Kaplan, & Palva, 2016).

## MATERIALS AND METHODS

### Data Acquisition and Preprocessing

We analyzed resting-state invasive stereotactic-electroencephalography (SEEG) recordings from a cohort of 27 epileptic patients (18 males, nine females; ages 16–21 years) and noninvasive magnetoencephalography (MEG) data recorded from 14 healthy subjects (seven males, seven females; ages 18–27 years).

Resting-state SEEG was collected for 10 min with eyes closed and without external disturbance using a 192-channel SEEG amplifier system (NIHON-KOHDEN NEUROFAX-110) at a sampling rate of 1 kHz. The number of electrode contacts along each penetrating shaft varied from eight to 15. These contacts were 2 mm long and 0.8 mm thick and had an intercontact distance of 1.5 mm (DIXI medical, Besancon, France). The anatomical positions and amounts of electrodes varied according to surgical requirements (Cardinale et al., 2013). We excluded from further analyses all contacts that were located within the epileptic focus or that exhibited epileptiform activity such as interictal spikes (Zhigalov et al., 2015). The MEG data were collected in a resting-state condition for 10 min while the subjects looked at a fixation point on the monitor screen. We recorded 306-channel (204 planar gradiometers and 102 magnetometers) MEG (Elekta Neuromag Ltd.) at a sampling rate of 600 Hz.

The SEEG study was approved by the Ethics Committee of the Niguarda “Ca’ Granda” Hospital, and the MEG study was approved by the Ethics Committee of Helsinki University Central Hospital. The subjects gave written informed consent for participation in research studies and for publication of the data. The studies were performed according to the Declaration of Helsinki.

### SEEG and MEG Preprocessing

The location of each SEEG electrode contact was extracted with submillimeter accuracy from postimplant cone-beam computerized tomography scans (Arnulfo, Narizzano, Cardinale, Fato, & Palva, 2015) and was subsequently coregistered to the Freesurfer (http://surfer.nmr.mgh.harvard.edu/) geometrical space. We used a “closest-white-matter” referencing scheme for the SEEG, in which electrode contacts in gray matter were referenced to the closest contacts in the underlying white matter (Arnulfo, Narizzano, et al., 2015). The MEG data were corrected for extracranial noise and for cardiac and eye blink artifacts by using the signal space separation method (Taulu, Simola, & Kajola, 2005) and independent-component analysis (Bell & Sejnowski, 1995), respectively. For cortical surface reconstructions, T1-weighted anatomical images were recorded with a 1.5-T magnetic resonance imaging (MRI) scanner (Siemens). The FreeSurfer software was used for automatic volumetric segmentation of the MRI data, surface reconstruction, flattening, and cortical parcellation. The MNE software (www.martinos.org/mne/) was used to create three-layer boundary element conductivity models and cortically constrained source models with fixed-orientation dipoles, and for computing the forward and inverse operators (Hamalainen & Ilmoniemi, 1994). The MEG sensor time series were filtered in multiple frequency bands using a broadband finite impulse response filter and Morlet’s wavelets. The filtered time series were inverse-transformed and collapsed into time series of 219 cortical parcels derived from the individual MRI scans (Daducci et al., 2012) by applying collapsing operators that maximized individual reconstruction accuracy (Korhonen, Palva, & Palva, 2014).

### Phase-Synchronization and Amplitude-Correlation Connectomes

To eliminate artificial interactions caused directly by volume conduction and signal mixing, we used linear-mixing-insensitive metrics for assessing interareal phase and amplitude interactions. Prior to the analyses, the time series were filtered using a bank of 31 Morlet’s wavelets with logarithmically spaced central frequencies ranging from 4 to 120 Hz. The phase synchronization between each pair of cortical parcels (MEG) or electrode contacts (SEEG) was estimated by the weighted phase lag index *q* (Vinck et al., 2011), as it is implemented in the Fieldtrip toolbox (Oostenveld, Fries, Maris, & Schoffelen, 2011):

$$q=\frac{{\left(\sum_{\omega }P_{xy}\left(\omega \right)\right)}^{2}-{\sum_{\omega }\left(P_{xy}\left(\omega \right)\right)}^{2}}{{\left(\sum_{\omega }\left|P_{xy}\left(\omega \right)\right|\right)}^{2}-\sum_{\omega }{\left(P_{xy}\left(\omega \right)\right)}^{2}}\text{,}$$ (1)

where *P*_*xy*_ is the imaginary part of the cross-spectral density

$$P_{xy}\left(\omega \right)\;=\;im\left(\sum_{m=1}^{N}E\left[x_{n}y_{n-m}\right]e^{-j\omega m}\right)\text{,}$$ (2)

and *x* and *y* are time series containing *N* samples; *E*[⋅] is the expectation operator, and *im*() denotes the imaginary part of a complex number.

The amplitude–amplitude correlations were assessed with the Pearson correlation coefficient. The narrow-band time series were orthogonalized for each pair of cortical parcels or electrode contacts by using linear regression (Brookes et al., 2012), and the correlation coefficient, *r*, was computed for the amplitude envelopes of the orthogonalized time series,

$$r\;=\left(\frac{E\left[\left(A_{X}-\mu_{A_{X}}\right)\left(A_{Y|X}-\mu_{A_{Y|X}}\right)\right]}{\sigma_{A_{X}}\sigma_{A_{Y|X}}}+\frac{E\left[\left(A_{X|Y}-\mu_{A_{X|Y}}\right)\left(A_{Y}-\mu_{A_{Y}}\right)\right]}{\sigma_{A_{X}|Y}\sigma_{A_{Y}}}\right)\;/\;2\text{,}$$ (3)

where *A*_*X*_, *A*_*Y*_ and *A*_*X*|*Y*_, *A*_*Y*|*X*_ are the amplitude envelopes of the original and orthogonalized time series, respectively; *µ* denotes the mean; and *σ* is the standard deviation.

The orthogonalization was defined as

$$Y_{|X}\;=\;Y-X\left(X^{+}Y\right)\text{,}$$ (4)

where *X* and *Y* are the narrow-band time series, and *X*^+^ denotes the pseudo-inverse of *X*. The orthogonalization *X*_|*Y*_ is done similarly (Brookes et al., 2012).

### Neuronal Avalanche Propagation Connectome

The neuronal avalanche propagation connectome was constructed by utilizing the concept of critical branching processes (Zapperi, Baekgaard Lauritsen, & Stanley, 1995), in which the activity in successive time bins propagates from one active group of neurons to another in an avalanche (Beggs & Plenz, 2003). Neuronal avalanches were detected in broadband-filtered (1–120 Hz), source-reconstructed MEG and SEEG time series that were normalized by subtracting the mean and dividing by the standard deviation. The time series were then transformed into binary point processes by detecting suprathreshold peaks above threshold *T* (Zhigalov et al., 2015). These binary sequences (or sequences of events) were converted into avalanche time series by summing the events across the channels in time bins *Δt*. A *neuronal avalanche* is defined as a cluster of events in successive time bins, where the beginning and end of the avalanche are defined by single time bins with no events (Figure 1A). The avalanche size distributions are typically fit well by a power law or a truncated power law, depending on the parameters *T* and *Δt* (Beggs & Plenz, 2003; Zhigalov et al., 2015). Using these avalanche data, we defined the avalanche propagation connectome to be constructed by the empirical probability with which suprathreshold peaks of neuronal activity would “transits” from the subset of channels in the first time bin to the subset of channels in the second time bin of each avalanche (Figures 1A and 1E). There are several approaches to assessing the network structure of activity propagation patterns using all time bins of an avalanche (Leleu & Aihara, 2015; Pajevic & Plenz, 2009). However, we assessed the propagation pathways by using only the first two bins of each avalanche (Beggs & Plenz, 2003) in order to sample the “source” and “target” parcels in the least ambiguous manner, because later bins might contain peaks that had originated from nonsuccessive bins. The avalanche propagation connectome was constructed for each subject separately from suprathreshold peaks of neuronal activity detected for the thresholds of two, three, and four *SD*s and a constant time bin width of 8 or 16 ms in MEG or SEEG, respectively (Figure 1B). The different time bin widths for MEG and SEEG were necessary to attain comparable scaling exponents of the neuronal avalanche sizes (Zhigalov et al., 2015). Goodness of fit was assessed by computing the Kolmogorov–Smirnov (KS) distance between the cumulative distribution functions (CDF) of the data and of a truncated power-law model (Klaus, Yu, & Plenz, 2011),

$$D_{KS}\;=\;\max\left|CDF_{\mathrm{data}}-CDF_{\mathrm{model}}\right|\text{.}$$ (5)

The corresponding *p*-value was estimated as follows:

$$p\;=\;\exp\left(-2{\left(\left(\sqrt{N^{2}/2N}+0.12\;+\frac{0.11}{\sqrt{N^{2}/2N}}\right)D_{KS}\right)}^{2}\right)\text{,}$$ (6)

where *N* denotes the number of samples.

The scaling exponents were estimated using the maximum likelihood method (Clauset, Shalizi, & Newman, 2009). Statistical comparison between the truncated power-law and exponential models has been done by applying the log-likelihood ratio test (Clauset et al., 2009; Klaus et al., 2011). A truncated power-law model is considered to outperform an exponential model if the log-likelihood ratio is positive and the difference between the model likelihoods is significant (*p* < 0.05). The probability functions of the truncated power-law and exponential distributions are expressed as

$$p_{t}\left(s\right)=\;C_{t}\;s^{-\alpha }e^{-\lambda s}\text{,}$$ (7)

$$p_{e}\left(s\right)=\;C_{e}e^{-\lambda s}\text{,}$$ (8)

where *p*(*s*) denotes the probability of observing an avalanche of size *s*, *C*_*t*_ and *C*_*e*_ are normalization constants, and *α* and *λ* are the parameters of the power-law and exponential functions, respectively.

The log-likelihood ratio between the two distributions is defined as

$$LLR\left(s\right)\;=\;l\left(\alpha ,\lambda |s\right)-l\left(\left\{\lambda \right\}|s\right)\text{,}$$ (9)

where *l*() denotes the log-likelihood functions, and *α* and *λ* are the parameters of the models.

The *p*-value for the *LLR* test is defined as follows:

$$p\;=\;\mathrm{erfc}\left(\frac{\left|LLR\right|}{\sqrt{2\pi \sigma^{2}}}\right)\text{,}$$ (10)

where $\sigma^{2}=\frac{1}{n}\overset{n}{\underset{i=1}{\sum }}{\left[\left(l\left(\{\alpha ,\lambda \}|s_{i}\right)-l(\{\alpha ,\lambda \}|s)/n\right)-\left(l\left(\{\lambda \}|s_{i}\right)-l(\{\lambda \}|s)/n\right)\right]}^{2}$.

### LRTC Connectomes

We used detrended fluctuation analysis (DFA) to assess the scaling exponents of the long-range temporal correlations (LRTCs; Linkenkaer-Hansen et al., 2001; J. M. Palva et al., 2013; Peng, Havlin, Stanley, & Goldberger, 1995). In DFA, the time series *X* is normalized to a zero mean and integrated over the samples, $Y(k)=\sum_{i=1}^{k}\left(X\left(i\right)-\langle X\rangle \right)$, then segmented into time windows of various sizes *τ*. Each segment of the integrated data is locally fitted to a linear function *U*_*τ*_, and the mean-squared residual *F*(*τ*) is computed,

$$F\left(\tau \right)\;=\;\sqrt{\frac{1}{N}\sum_{k=1}^{N}{\left[Y_{\tau }\left(k\right)-U_{\tau }\left(k\right)\right]}^{2}}\text{,}$$ (11)

where *N* is the number of samples in segment *τ*.

The scaling exponent *η* is defined as the coefficient of the linear regression of the function *F*(*τ*) plotted in double logarithmic coordinates. The optimal fitting range, in terms of the linearity of the slope, was limited to 3–300 s for the present dataset.

The LRTC connectome was computed for the amplitude envelopes of the wavelet-filtered time series using the Pearson correlation coefficient at the group level between the exponents of each cortical parcel *i* (*η*_*i*_) and *j* ($\eta_{j}^{\#})$, where $\eta_{j}^{\#}$ was assessed with the time series of parcel *j* orthogonalized to the parcel *i* (Brookes et al., 2012),

$$A_{ij}\;=\;\frac{E\left[\left(\eta_{i}-\mu_{\eta_{i}}\right)\left(\eta_{j}^{\#}-\mu_{\eta_{j}^{\#}}\right)\right]}{\sigma_{\eta_{i}}\sigma_{\eta_{j}^{\#}}}\;\text{.}$$ (12)

As in the estimation of amplitude–amplitude correlations, orthogonalization was used to eliminate the pairwise effects of linear signal mixing on the LRTC estimates, and hence to allow for assessment of the relationship between local cortical scaling exponents without artificial coupling of these regions (Blythe, Haufe, Muller, & Nikulin, 2014).

### Surrogates

To assess the statistical significance of the results, we generated comparable surrogate data and applied the analyses above to these data. The surrogate time series were derived from the original ones so that the signal of each channel was circularly shifted by a random number of samples (Zhigalov et al., 2015). This approach provides more accurate statistical inference for neuronal avalanches than does the conventional phase-shuffling method (Prichard & Theiler, 1994). The circular shift breaks spatial correlations but essentially fully preserves the temporal autocorrelation structure. For SEEG, the surrogate data were obtained by circular-shifting the white-matter-referenced gray-matter recordings and used as is. For MEG, the surrogate data were obtained by first circular-shifting the original source-reconstructed MEG parcel time series and then forward and re-inverse modeling these data (Korhonen et al., 2014). This approach is essentially equivalent to the one proposed by Shahbazi and colleagues (Shahbazi, Ewald, Ziehe, & Nolte, 2010) and accurately reconstructed the net residual signal mixing present after the MEG measurement (forward modeling) and after the partial separation of the original cortical sources achieved by source modeling (inverse modeling).

### Control Analyses for the Avalanche Propagation Connectome

To assess whether the avalanche propagation and phase-synchronization or amplitude-correlation connectomes can be estimated independently, we performed several lines of control simulations. We assessed the impacts of suprathreshold events—that is, neuronal avalanches—on FC in MEG using two approaches. In the first approach (see Figure 4A), we deleted all suprathreshold data segments above three *SD*s and recomputed the phase and amplitude connectomes (see Figure 4A, solid lines). This deletion was carried out separately for each parcel pair in these connectomes by using logical disjunction (i.e., the OR operation) of the avalanche segments in these parcels. We also recomputed the phase and amplitude connectomes for the complement of these data—that is, by using only the suprathreshold segments (see Figure 4A, dashed lines). The suprathreshold, subthreshold, and original functional phase/amplitude connectomes were compared with the avalanche propagation connectome using the edge similarity index (Eq. 14 below). Any statistical difference between the edge similarities at multiple frequencies was assessed with the KS test.

In the second approach (see Figure 4B), we swapped all time windows of the suprathreshold avalanche segments above three *SD*s between randomly assigned parcel pairs. This operation thus randomly reordered the avalanche propagation connectome. Because the segments were only partially overlapping between the parcel time series, we again used disjunction to combine avalanche segments within the pairs of parcels. After the segment swaps, the time series were refiltered to account for the filter-related temporal spreading of the large-amplitude avalanche events. Using these data, we then recomputed the phase and amplitude connectomes and assessed their edge similarity with the original avalanche connectome (see Figure 4B, solid lines). To assess how independently the avalanches and FC can be measured, we also reanalyzed the avalanche propagation patterns from these data and compared this swapped avalanche propagation connectome with the swapped phase and amplitude connectomes using the edge similarity index (see Figure 4B, dashed lines).

### Estimation of the Branching Ratio

The branching ratio (*p*) was estimated by computing the ratio of the number of events in the second time bin to the number of events in the first time bin of a neuronal avalanche (Beggs & Plenz, 2003; Shriki et al., 2013).

$$p\;=\;\frac{1}{N}\sum_{i=1}^{N}\frac{n_{i}^{\left(2\right)}}{n_{i}^{\left(1\right)}}\text{,}$$ (13)

where $n_{i}^{(1)}$ and $n_{i}^{(2)}$ are the numbers of events in the first and second time bins of the *i*th avalanche, respectively; *N* is the total number of avalanches.

### Phase DFA

In addition to classical avalanche and amplitude LRTC measures, we characterized criticality in the LRTCs with moment-to-moment fluctuations of interareal phase synchrony (Botcharova et al., 2014). The phase time series between pairs of cortical parcels (MEG) or contacts (SEEG) were extracted from the complex-valued narrow-band-filtered data (4–120 Hz). DFA (see the LRTC Connectome section above) was applied to the first time derivatives of the phase differences. The resulting connectome represented the LRTC scaling exponents of phase synchrony between each pair of cortical parcels.

### Control Analysis for the LRTC Connectome

To assess whether the connection strengths (connectome values) have a specific frequency profile that might explain the profile of connectome similarity, we estimated the connection strengths for the phase, amplitude, and LRTC connectomes for the entire range of frequencies (4–120 Hz). The connection strengths were estimated as a mean for the connectome (adjacency matrix) for each frequency (see Figure 6).

### Edge and Subgraph Similarity Indices

The *edge similarity* index was defined as the Pearson correlation between two adjacency matrices (connectomes),

$$h_{e}\;=\;\frac{E\left[\left(A-\mu_{A}\right)\left(B-\mu_{B}\right)\right]}{\sigma_{A}\sigma_{B}}\text{,}$$ (14)

where *A* and *B* are inflated adjacency matrices (column vectors).

The modular structure of the connectomes was detected using agglomerative hierarchical clustering (Bellec, Rosa-Neto, Lyttelton, Benali, & Evans, 2010). The number of clusters per hemisphere, *K* = 7, provided a reasonable division of the connectomes into functional modules. As a result of clustering, each cortical parcel *n* (*N* = 219) was associated with a unique subgraph (or cluster) identity (*I*_*n*_ ∈ [1, K]). The *subgraph similarity* index was defined as the Jaccard similarity coefficient between matched subgraph identities,

$$h_{s}=\frac{I_{A}\cap I_{B}}{I_{A}\cup I_{B}}\text{,}$$ (15)

where *I*_*A*_ and *I*_*B*_ are the vectors of cluster identifies computed for the *A* and *B* adjacency matrices, respectively.

### Consensus Mapping of Subgraph Identities

Consensus mapping was applied to assess the shared subgraphs of the connectomes. The subgraph identities (see the previous section) of all four connectomes at a certain frequency (e.g., 10 Hz; see Figures 7E and 7G) were iteratively reordered to maximize overlap between the subgraphs. To do this, the vectors of subgraph identities of size [*N* × 1] were transformed into binary arrays of size [*N* × *K*], where *N* is the number of parcels (*N* = 219) and *K* is the number of subgraph identities (*K* = 7). Nonzero items of the array represented associations between a parcel (row) and its subgraph identity (column), so that each row contained only one nonzero item. The binary arrays of subgraph identities were assessed for all four connectomes. The columns of these arrays were iteratively reordered to maximize the sum of the element-wise product between the four arrays. The reordered binary arrays were then wrapped back to the subgraph identity vectors and visualized (e.g., Figures 7E and 7G).

## ACKNOWLEDGMENTS

This study was supported by the Academy of Finland, Grant Nos. 253130 and 256472 (to J.M.P.) and 1126967 (to S.P.); by the Helsinki University Research Funds and European Union Seventh Framework Programme (FP7/2007-2013) under Grant Agreement No. 29 604102 (Human Brain Project); and by the Italian Ministry of Health, Targeted Research Grant No. RF-2010-2319316 (to L.N.).

## AUTHOR CONTRIBUTIONS

Alexander Zhigalov: Data curation; Formal analysis; Methodology; Software; Visualization; Writing – original draft; Writing – review & editing Gabriele Arnulfo: Data curation; Methodology; Validation Lino Nobili: Data curation Satu Palva: Project administration; Resources J. Matias Palva: Conceptualization; Funding acquisition; Project administration; Resources; Supervision; Writing – review & editing

## TECHNICAL TERMS

Functional connectivity: Defined as the correlation between neurophysiological time series
Scale-free dynamics: Any fractally self-similar—that is, power-law scaled—dynamics of a system
Neuronal avalanche: A cascade of neuronal activity with power-law size and lifetime distributions
Long-range temporal correlations: Functional correlations characterized by power-law decay of the autocorrelation function
Criticality: The state of a dynamical system at a boundary between two qualitatively different types of dynamics
Neuronal oscillations: Transiently rhythmic neuronal population activities
Resting-state: An experimental paradigm in which the participant does not receive any stimulation or task
SEEG: An invasive, presurgical technique in which local field potentials are recorded intracerebrally to identify the areas where epileptic seizures originate
Critical branching: A stochastic process characterized by a nearly constant mean population size—that is, without dying out or explosive growth
